# What is the evidence for the use of low-pressure pneumoperitoneum? A systematic review

**DOI:** 10.1007/s00464-015-4454-9

**Published:** 2015-08-15

**Authors:** Denise M. D. Özdemir-van Brunschot, Kees C. J. H. M. van Laarhoven, Gert-Jan Scheffer, Sjaak Pouwels, Kim E. Wever, Michiel C. Warlé

**Affiliations:** Division of Vascular and Transplant Surgery, Department of Surgery, Radboud University Medical Center, Geert Grooteplein-Zuid 10, PO Box 9101, 6500 HB Nijmegen, The Netherlands; Department of Anesthesiology, Radboud University Medical Center, Nijmegen, The Netherlands; Systematic Review Centre for Laboratory Animal Experimentation (SYRCLE), Radboud University Medical Center, Nijmegen, The Netherlands

**Keywords:** Laparoscopy, Pneumoperitoneum, Low pressure, Pain, Perioperative conditions

## Abstract

**Background:**

Laparoscopic surgery has several advantages when compared to open surgery, including faster postoperative recovery and lower pain scores. However, for laparoscopy, a pneumoperitoneum is required to create workspace between the abdominal wall and intraabdominal organs. Increased intraabdominal pressure may also have negative implications on cardiovascular, pulmonary, and intraabdominal organ functionings. To overcome these negative consequences, several trials have been performed comparing low- versus standard-pressure pneumoperitoneum.

**Methods:**

A systematic review of all randomized controlled clinical trials and observational studies comparing low- versus standard-pressure pneumoperitoneum.

**Results and conclusions:**

Quality assessment showed that the overall quality of evidence was moderate to low. Postoperative pain scores were reduced by the use of low-pressure pneumoperitoneum. With appropriate perioperative measures, the use of low-pressure pneumoperitoneum does not seem to have clinical advantages as compared to standard pressure on cardiac and pulmonary function. Although there are indications that low-pressure pneumoperitoneum is associated with less liver and kidney injury when compared to standard-pressure pneumoperitoneum, this does not seem to have clinical implications for healthy individuals. The influence of low-pressure pneumoperitoneum on adhesion formation, anastomosis healing, tumor metastasis, intraocular and intracerebral pressure, and thromboembolic complications remains uncertain, as no human clinical trials have been performed. The influence of pressure on surgical conditions and safety has not been established to date. In conclusion, the most important benefit of low-pressure pneumoperitoneum is lower postoperative pain scores, supported by a moderate quality of evidence. However, the quality of surgical conditions and safety of the use of low-pressure pneumoperitoneum need to be established, as are the values and preferences of physicians and patients regarding the potential benefits and risks. Therefore, the recommendation to use low-pressure pneumoperitoneum during laparoscopy is weak, and more studies are required.

Based on experiments in dogs by Georg Kelling, Hans Christian Jacobaeus was the first to perform a laparoscopic procedure in humans in 1910 [1, 2]. Insufflation of air into the peritoneal cavity created working space between the abdominal wall and the intraabdominal organs. Until the 1960s, the physiological consequences of increased intraabdominal pressure by gas insufflation were poorly understood. In 1966, Kurt Semm introduced an automatic insufflation device capable of monitoring intraabdominal pressure, thereby improving the safety of laparoscopy [3]. Today, intraabdominal pressure is traditionally set at a routine pressure of 12–15 mmHg [4]. Bearing in mind the potential negative impact of pneumoperitoneum (PNP) on cardiopulmonary function and the positive impact on postoperative pain, international guidelines recommend that the use of “the lowest intraabdominal pressure allowing adequate exposure of the operative field rather than a routine pressure” should be used [5]. In literature, low-pressure PNP is generally defined as an intraabdominal pressure of 6–10 mmHg [6–9]. However, in daily clinical practice, usually the intra-abdominal pressure is set at 12–14 mmHg, and for gynecological laparoscopic procedures, sometimes even higher pressures are used. In this systematic review, we will address the risks and benefits of low- versus standard-pressure PNP.

## Materials and methods

This review was performed in accordance with the PRISMA guidelines. The MEDLINE, EMBASE, and Cochrane databases were systematically searched from January 1, 1995 to September 1, 2014, and the search strategy is provided in Table 1. Two authors (DÖ and SP) independently confirmed the eligibility of the studies. To identify other relevant randomized controlled clinical trials, the references of the identified trials and cross references were searched. Only randomized clinical trials (RCT) and cohort studies comparing low- versus standard-pressure PNP were included.

**Table 1:** Search Strategy

Database	Search strategy
PubMed	(laparoscop* OR coelioscop* OR celioscop* OR peritoneoscop*) AND (pneumoperitoneum OR pneumoperitoneum, Artificial[MeSH] OR insufflation OR insufflation[MeSH]) AND (randomized controlled trial[pt] OR controlled clinical trial[pt] OR randomized [tiab] OR randomly[tiab] OR trial[tiab])
EMBASE	1. (laparoscop* or coelioscop* or celioscop* or peritoneoscop*).af 2. exp Laparoscopic Surgery/ 3. 1 or 2 4. (pneumoperitoneum or insufflation).af 5. exp Pneumoperitoneum/ 6. 4 OR 5 7. 3 AND 6 8. exp RANDOMIZED CONTROLLED TRIALS/ 9. 7 and 8
CENTRAL	1. Laparoscop* OR coelioscop* OR celioscop* OR peritoneoscop* 2. MeSH description Pneumoperitoneum, Artificial, explode all trees 3. MeSH description Insufflation, explode all trees 4. 1 OR 2 OR 3

The following characteristics were extracted: author, year of publication, country of hospital, study design, total number of patients, total number of patients in each experimental arm, mean age and standard deviation (SD), gender, mean body mass index (BMI) (SD), type of laparoscopic procedure, and definitions of low and standard pressures.

Outcome measures included: postoperative pain and analgesia consumption, pulmonary and cardiac function, liver and kidney function, thromboembolic complications, adhesion formation, anastomosis healing, intracranial and intraocular pressure, tumor growth and metastases and perioperative conditions, complications, and conversion to open procedure. When enough data were available, a meta-analysis was performed. Meta-analysis was performed using Review Manager (version 5.2, the Cochrane Collaboration, Oxford, UK). Data were pooled using random-effects model. Continuous data were expressed as mean difference, and consistency was measured with I^2^.

Quality assessment of randomized controlled trials was performed using the Cochrane Collaboration’s tool for assessing risk of bias [10] by two authors (DÖ and SP) independently. The quality of non-randomized trials was assessed with the Newcastle–Ottawa Rating scale [11]. Two stars were awarded when body mass index (BMI), age, and gender were comparable. The follow-up had to be at least 3 days to score one point on the “follow-up” item. This way, major complications were not missed due to a too short follow-up period. The quality of evidence and strength of recommendation were assessed according to the GRADE approach [12].

## Results

**Fig. 1:**
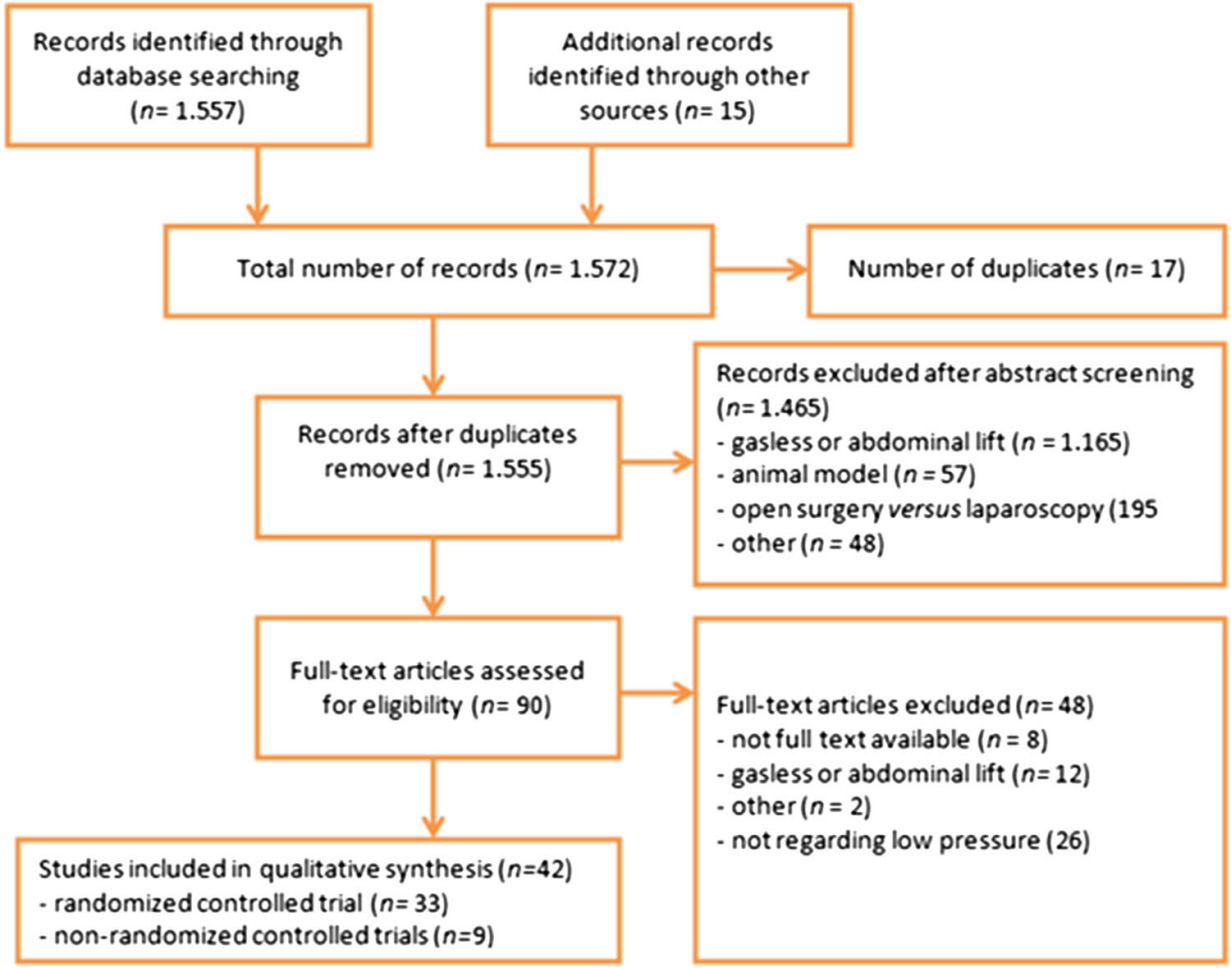
Flowchart of study search

**Fig. 2:**
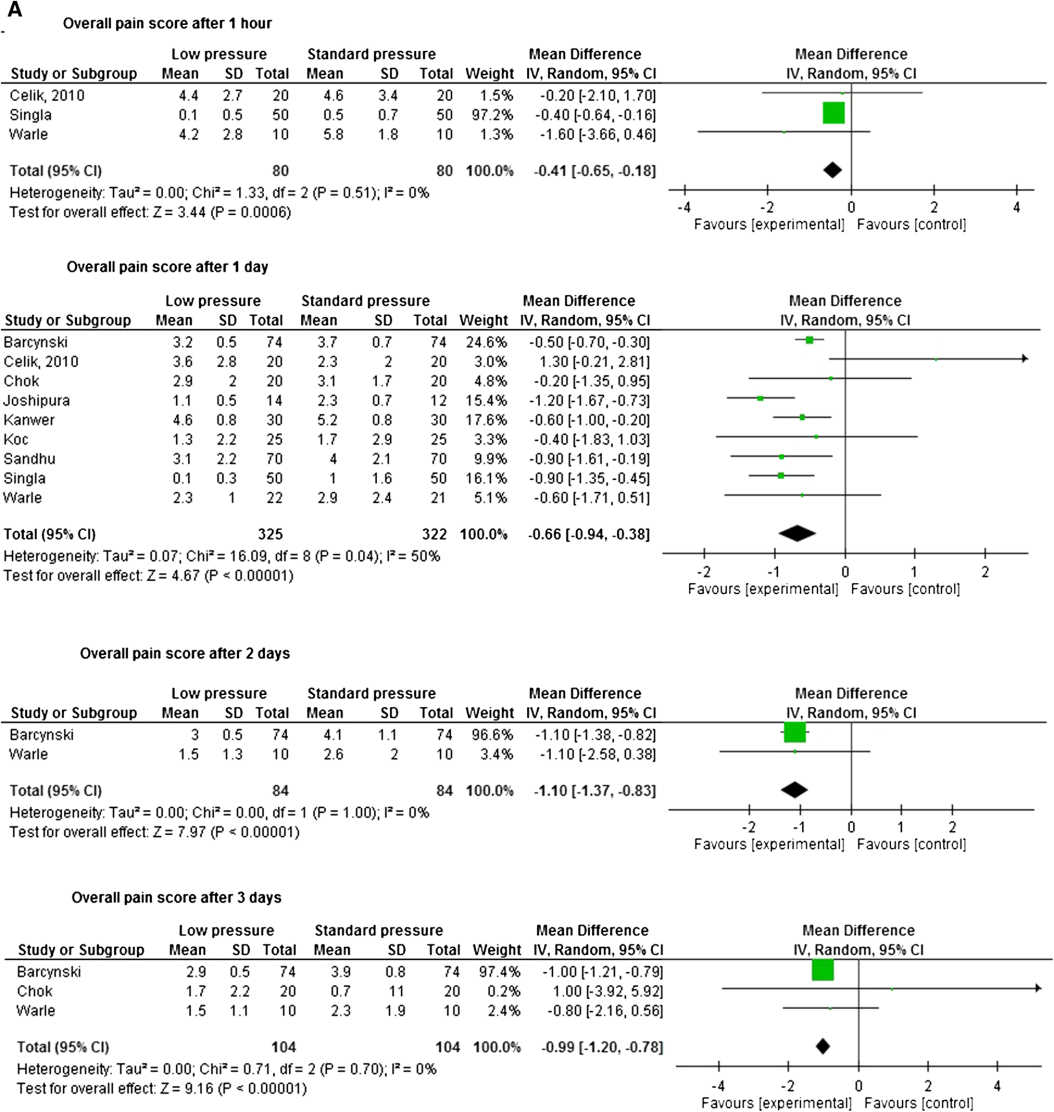

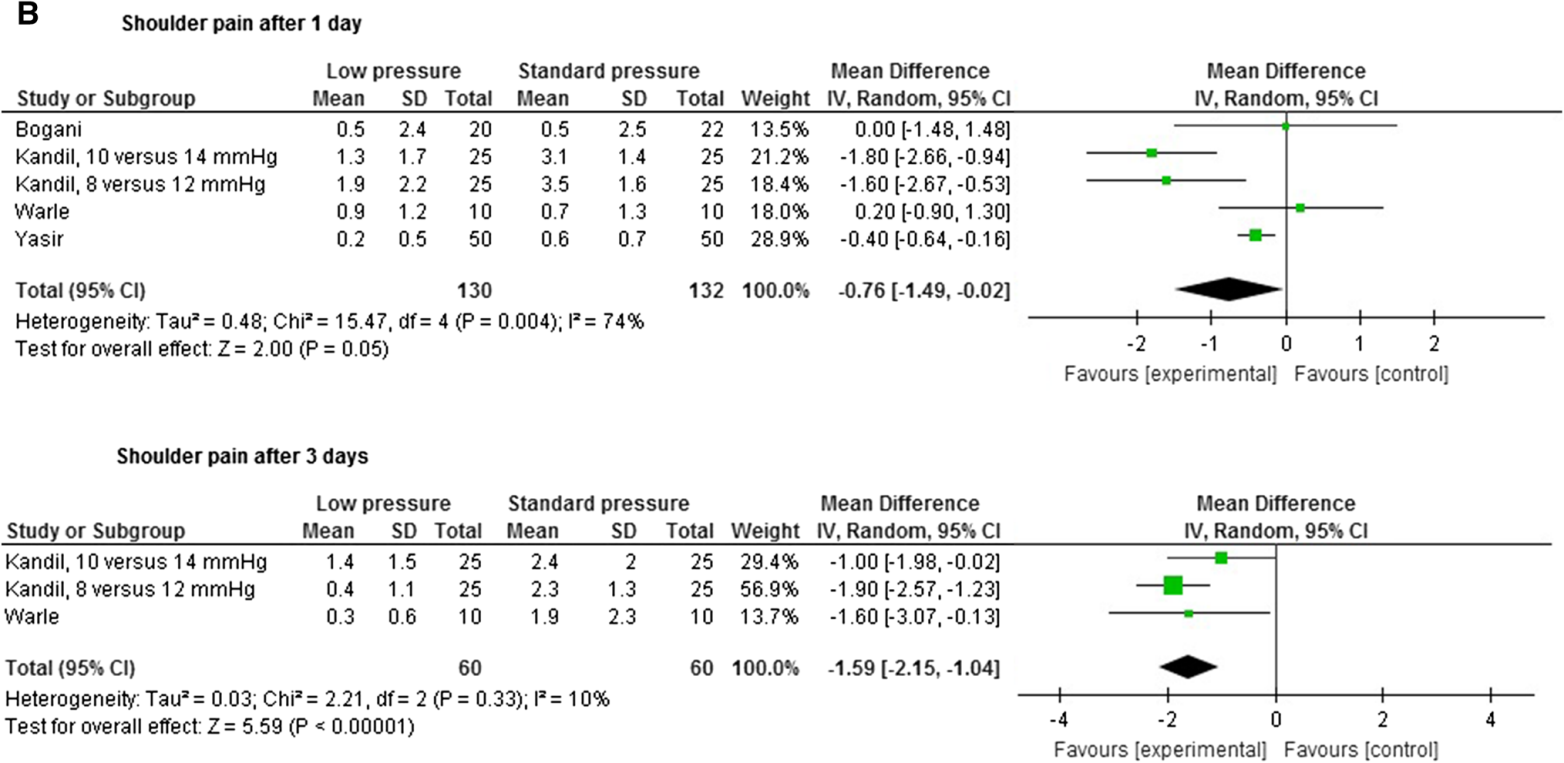
**A** Meta-analysis of overall pain. **B** Shoulder pain

Of the 1572 papers identified at the initial search, 42 were included after abstract and full-text screening (Fig. 1). Characteristics of the included studies are shown in Tables 2 and 3. The quality assessment of the available evidence using the Cochrane Collaboration’s tool and the Newcastle–Ottawa scale for assessing risk of bias is shown in Tables 4 and 5; in general, the quality of the included studies was low or unclear [10]. For five studies, information that Gurusamy et al. obtained by contacting the authors was reused to supplement the quality assessment. An overview of the results, including quality of evidence according to GRADE, is provided in Table 7 (Fig. 2).

**Table 2:** Characteristics of human randomized controlled trials

First author	Year of publication	Country	Pressure	Procedure	Number of patients
Barczynski [2]	2002	Poland	7 versus 12	LC	74 versus 74
Basgul [13]	2004	Turkey	10 versus 14–15	LC	11 versus 11
Bogani [14]	2014	Italy	8 versus 12	LH	20 versus 22
Celik [15]	2004	Turkey	8 versus 10 versus 12 versus 14 versus 16	LC	20 versus 20 versus 20 versus 20 versus 20
Celik [16]	2010	Turkey	8 versus 12 versus 14	LC	20 versus 20 versus 20
Chok [17]	2006	China	7 versus 12	LC	20 versus 20
Dexter [18]	1998	UK	7 versus 15	LC	10 versus 10
Ekici [19]	2009	Turkey	7 versus 15	LC	20 versus 32
Emad Esmat [9]	2006	Egypt	10 versus 14	LC	34 versus 37
Eryilmaz [6]	2011	Turkey	10 versus 14	LC	20 versus 23
Gupta [20]	2013	India	8 versus 14	LC	50 versus 51
Hasukic [21]	2005	Bosnia-Herzegovina	7 versus 14	LC	25 versus 25
Ibraheim [7]	2006	Saudi Arabia	6–8 versus 12–14	LC	10 versus 10
Joshipura [22]	2009	India	8 versus 12		
Kandil [23]	2010	Egypt	8 versus 10 versus 12 versus 14	LC	25 versus 25 versus 25 versus 25
Kanwer [24]	2009	India	10 versus 14	LC	27 versus 28
Karagulle [25]	2008	Turkey	8 versus 12 versus 15	LC	14 versus 15 versus 15
Koc [26]	2005	Turkey	10 versus 15	LC	25 versus 25
Morino [27]	1998	Italy	10 versus 14	LC	10 versus 22
Perrakis [28]	2003	Greece	8 versus 15	LC	20 versus 20
Polat [29]	2003	Turkey	10 versus 15	LC	12 versus 12
Sandhu [30]	2009	Thailand	7 versus 14	LC	70 versus 70
Sarli [31]	2000	Italy	9 versus 13	LC	46 versus 44
Schietroma [8]	2013	Italy	6–8 versus 12–14	LNF	33 versus 35
Sefr [32]	2003	Czech Republic	10 versus 15	LC	15 versus 15
Singla [33]	2014	India	7–8 versus 12–14	LC	50 versus 50
Sood [34]	2006	India	8–10 versus 15	LA	5 versus 4
Topal [35]	2011	Turkey	10 versus 13 versus 16	LC	20 versus 20 versus 20
Torres [36]	2009	Poland	6–8 versus 10–12	LC	20 versus 20
Umar [37]	2011	India	8–10 versus 11–13 versus 14	LC	Unclear
Vijayaraghavan [38]	2014	India	8 versus 12	LC	22 versus 21
Wallace [39]	1997	UK	7.5 versus 15	LC	20 versus 20
Warlé [40]	2013	the Netherlands	7 versus 12	LDN	10 versus 10
Yasir [41]	2012	India	8 versus 14	LC	50 versus 50

*LA* laparoscopic adrenalectomy, *LC* laparoscopic cholecystectomy, *LDN* laparoscopic donor nephrectomy, *LH* laparoscopic hysterectomy, *LNF* Laparoscopic nissen fundoplication

**Table 3:** Characteristics of non-randomized trials

First author	Year of publication	Country/state	Pressure	Procedure	Number of patients
Atila [42]	2009	Turkey	N/A	LC	40
Davides [43]	1999	UK	7 versus 10.6	LC	50 versus 77
Hawasli [44]	2003	USA	10 versus 15	LDN	25 versus 25
Kamine [45]	2014	USA	N/A	LA VERSUSP	9
Kovacs [46]	2012	Hungary	8 versus 13	LDN	44 versus 26
Matsuzaki [47]	2011	France	8 versus 12	LH	32 versus 36
Park [22]	2012	Korea	10 versus 15	LCol	30
Rist [48]	2001	Germany	10 versus 15	L	10
Schwarte [38]	2004	Germany	8 versus 12	DL	16

*DL* diagnostic laparoscopy, *L* laparoscopy of the lower abdomen, *LA VSP* laparoscopy-assisted ventriculoperitoneal shunt placement, *LC* laparoscopic cholecystectomy, *LCol* laparoscopic colectomy, *LDN* laparoscopic donor nephrectomy, *LH* laparoscopic hysterectomy

**Table 4:** Quality assessment of included human randomized controlled trials according to Cochrane

First author	Random sequence	Allocation concealment	Blinding	Incomplete outcome	Selective reporting
Barczynski [2]	Low	Low	Unclear	Unclear	Unclear
Basgul [13]	Unclear	Unclear	Unclear	Unclear	Unclear
Bogani [14]	Low	Unclear	Unclear	Low	Low
Celik [15]	Unclear	Unclear	Unclear	Unclear	Unclear
Celik [16]	Low	Unclear	Unclear	Low	Unclear
Chok [17]	Low	Low	Unclear	Low	Unclear
Dexter [18]	Unclear	Unclear	Unclear	Low	Unclear
Ekici [19]	Unclear	Unclear	Unclear	Unclear	High
Emad Esmat [9]	Low	Unclear	Unclear	Unclear	Unclear
Eryilmaz [6]	Unclear	Unclear	Unclear	Low	Unclear
Gupta [20]	Low	Low	Unclear	Low	Unclear
Hasukic [21]	Low	Low	Unclear	Unclear	Unclear
Ibraheim [7]	Unclear	Low	Unclear	Unclear	Unclear
Joshipura [22]	Unclear	Low	Low	Unclear	Unclear
Kandil [23]	Unclear	Unclear	Unclear	Low	Unclear
Kanwer [24]	Low	Unclear	Unclear	Unclear	Unclear
Karagulle [25]	Unclear	Unclear	Unclear	Low	Unclear
Koc [26]	Unclear	Low	Unclear	Low	Unclear
Morino [27]	Unclear	Unclear	Unclear	Unclear	Unclear
Perrakis [28]	Low	Unclear	Unclear	Unclear	Unclear
Polat [29]	Unclear	Unclear	Unclear	Unclear	Unclear
Sandhu [30]	Unclear	Unclear	Unclear	Low	Unclear
Sarli [31]	Unclear	Unclear	Low	Low	Unclear
Schietroma [8]	Unclear	Unclear	Unclear	Unclear	Unclear
Sefr [32]	Low	Unclear	Unclear	Low	Unclear
Singla [33]	Low	unclear	Unclear	Low	Unclear
Sood [34]	Low	Unclear	Unclear	Low	Unclear
Topal [35]	Unclear	Unclear	Unclear	Low	Unclear
Torres [36]	Unclear	Unclear	Unclear	Low	Low
Umar [37]	Unclear	Unclear	Unclear	High	Unclear
Vijayaraghavan [38]	Low	Low	Low	Low	Unclear
Wallace [39]	Low	Unclear	Unclear	Low	Unclear
Warlé [40]	Unclear	Low	Low	Low	Unclear
Yasir [41]	Unclear	Unclear	Unclear	High	Unclear

**Table 5:** Quality assessment of included non-randomized trials according to Newcastle–Ottawa

First author	Selection				Comparability	Outcome			Total
	Representiveness	Selection	Ascertainment	Demonstration		Assessment	Follow-up	Adequacy	
Atila [42]	*	*	*	*	**			*	7
Davides [43]		*	*	*				*	4
Hawasli [44]	*	*	*	*	**			*	7
Kamine [45]	*	*	*		**			*	6
Kovacs [46]	*	*	*	*	**			*	7
Matsuzaki [49]		*	*	*	*			*	5
Park [50]	*	*	*	*	**			*	7
Rist [48]	*	*	*	*	**			*	7
Schwartz [51]	*	*	*	*	**			*	7

### Pain

A Cochrane review performed by Gurusamy et al. in 2009 regarding elective and emergency laparoscopic cholecystectomy showed decreased pain scores during the early postoperative phase. Nevertheless, definite conclusions could not be drawn from this meta-analysis since most studies were at high risk of bias [52]. In the recently updated Cochrane review, pain scores were not included, and it was stated that “pain scores are unvalidated surrogate outcomes for pain in people undergoing laparoscopic cholecystectomy and several Cochrane systematic reviews have demonstrated that pain scores can be decreased with no clinical implications in people undergoing laparoscopic cholecystectomy” [53]. However, in literature there is evidence that a reduction of 1.0–1.5 points on an 0–10 pain scale is a clinically relevant difference [54–57]. In four studies, the effects of low-pressure PNP were assessed in a blinded fashion [22, 31, 38, 40]. In three studies, overall pain scores were assessed and in two studies, and a clinically relevant difference was found at postoperative day 1. From the patients’ perspective, the duration of reduction in postoperative pain is also important. The only blinded study comparing postoperative pain longer than 24 h after surgery is the study by Warlé et al. [40]. In this study, a difference of 0.8 in overall pain score on an 0–10 scale 3 days after surgery was observed. Regarding shoulder pain, in two studies this parameter was assessed, in one study a difference of approximately two points was found up to postoperative day 1 [26], while in the other study mean pain scores of 0.7 and 0.9 were observed [40, 58].

Randomized controlled trials comparing non-cholecystectomy procedures (i.e., laparoscopic donor nephrectomy and laparoscopic gynecologic procedures) also suggests that low-pressure PNP is associated with less postoperative pain [14, 40, 44, 46, 47, 59].

In Table 6a, b, an overview of overall pain scores and shoulder pain in low pressure versus standard pressure is shown. Meta-analysis of pain scores at different time point shows that overall pain was significant lower in the low-pressure group; however, this difference was only clinically relevant after 2 and 3 days. After 1 and 3 days, shoulder pain was significantly lower for the low-pressure group, and this difference was clinically significant after 3 days.

**Table 6:** Assessment of (a) overall postoperative pain, (b) shoulder pain

First author	1 h		Day 1		Day 2		Day 3	
	Low pressure	Standard pressure	Low pressure	Standard pressure	Low pressure	Standard pressure	Low pressure	Standard pressure
*(a) Overall postoperative pain*								
Barcynski [2]			3.2	3.7	3.0	4.1	2.9	3.9
Celik [16]	4.4	4.6	3.6	2.3				
Chok [17]			2.9	3.1			1.7	0.7
Joshipura [22]			1.1	2.3				
Kanwer [24]			4.6	5.2				
Koc [26]			1.3	1.7				
Sandhu [30]			3.1	4.0				
Singla [33]	0.1	0.5	0.1	1.0				
Vijavaraghavan [38]			1	3				
Warlé [40]	4.2	5.8	2.3	2.9	1.5	2.6	1.5	2.3
*(b) Shoulder pain*								
Bogani [14]	0.8	5.0	0.5	0.5				
Esmat [9]			1.3	2.5			0.2	0.3
Kandil [23]			1.3 and 1.9	3.1 and 3.5			0.4 and 1.4	2.3 and 2.4
Warlé [40]	0.0	0.2	0.9	0.7	0.6	1.4	0.3	1.9
Yasir [41]			0.2	0.6				

### Pulmonary function

Despite the fact that in one RCT, pulmonary compliance was significantly compromised in the standard-pressure group when compared to low-pressure PNP [50], end tidal CO_2_, pCO_2_, oxygen saturation, pO_2_ and blood gas analyses, including pH, bicarbonate or base excess, were comparable [22, 32, 38–40]. Postoperative pulmonary function tests were evaluated by three RCTs, and no significant differences in pulmonary function tests were observed [22, 25, 38]. No RCTs comparing low- versus standard-pressure PNP in patients with pulmonary comorbidities are performed.

### Cardiac function

When comparing cardiac function in low- versus standard-pressure PNP in human trials, most studies comparing heart frequency, cardiac index, and mean arterial pressure did not observe a significant difference [6, 18, 19, 34, 60]. These findings also seem to be applicable for ASA III and IV patients, as Koivusalo et al. [60] compared hemodynamic, renal, and liver parameters in ASA III and IV patients in low-pressure versus standard-pressure PNP and found no significant differences. However, it should be noted that not all studies demonstrated consistent results [37]: Umar et al. observed a significant decrease in mean heart rate and mean systolic blood pressure.

### Liver function

Two studies observed a pressure-dependent decrease in hepatic blood flow and enzyme elevations of AST and ALT [21, 27], whereas postoperative bilirubin, γ-GT, and ALP were not or slightly elevated [21, 61–63]. Eryilmaz et al. [6] used indocyanine green elimination tests (ICG-PDR) as a parameter for liver function. In their trial, a significant decrease in ICG-PDR values in the standard pressure (14 mmHg) PNP was observed when compared to the low-pressure group (10 mmHg). In none of the trials, persistent elevation of liver enzymes or liver failure was observed.

### Renal function

Human trials comparing renal function during and after low-pressure compared to standard-pressure PNP are scarce. In two RCTs, urine output was lower in the standard-pressure group, but no changes in postoperative creatinine could be demonstrated [40, 44]. Preoperative volume loading before and during PNP can help maintaining renal perfusion [64]. With the exception of a few case reports [65–67], in the postoperative phase, serum creatinine levels, creatinine clearance, and urine output returned to normal in all patients.

### Thromboembolic complications

The difference in the incidence of deep venous thrombosis or pulmonary embolism during low or normal intra-abdominal pressure has not been described. However, four studies indirectly evaluated the risk of thromboembolic complications. First, Ido et al. [68] demonstrated that blood flow velocity in the femoral vein was significantly reduced during abdominal insufflation, and there was a significant difference when using 5 or 10 mmHg intra-abdominal pressure. Topal et al. [35] assessed different thromboelastographic parameters, e.g., reaction time, maximum amplitude, α-angle, and K time, in low (10 mmHg) versus standard (13 mmHg) and high intra-abdominal pressure (16 mmHg). All parameters were comparable to preoperative values in the 10 mmHg group and the 13 mmHg group. Two other randomized controlled trials observed no significant differences in diameter of the common iliac vein when pressure was increase from, respectively, 10 to 15 and 8 to 12 mmHg [22, 48].

### Adhesions

No human trials have been performed comparing adhesion formation in low-pressure versus standard-pressure PNP.

### Anastomosis healing

No human randomized controlled trials comparing anastomotic leakage in low-pressure versus standard-pressure PNP have been performed. In one study, low- versus standard-pressure was compared in colorectal procedures; however, the incidence of anastomotic leakage was not recorded [50].

### Intracranial pressure

Kamine et al. [45] compared intracranial pressure at different intra-abdominal pressures in nine patients undergoing laparoscopy-assisted ventriculoperitoneal shunt placement. They observed a pressure-dependent increase after abdominal insufflation, and maximum intracranial pressure was 25 cm H_2_O at an insufflation pressure of 15 mmHg. No trials comparing intracranial pressure in low-pressure versus standard-pressure PNP in humans have been performed.

### Intraocular pressure

Although clinical trials in humans have shown that laparoscopic procedures are associated with increased intraocular pressure when compared to open procedures, it remains unclear whether this can solely by attributed to increased intra-abdominal pressure; type of anaesthesia and position of the patient probably also play an important role [69–71]. No clinical trials in humans have been performed comparing intraocular pressure in low- versus normal-pressure PNP.

### Tumor growth and metastases

Data from human trials are lacking.

### Peri- and postoperative inflammatory response

In five studies, the inflammatory response in low- versus standard-pressure PNP are compared [8, 13, 28, 36, 38]. Schietroma et al. [8] observed a significant decrease in interleukin (IL)-1, IL-6, and C-reactive protein (CRP); however, this could not be confirmed in the studies performed by Perrakis, Torres, and Vijayaghavan et al. [28, 36, 38]. Basgul et al. [13] observed a significant lower increase in IL-6 up to 24 h after surgery, but higher levels of IL-2 during low-pressure PNP.

### Quality of surgical conditions

Because the use of low-pressure PNP might decrease the effective working space, one of the major concerns is risk of intra-abdominal organ injury. Perioperative surgical conditions are reported in three randomized controlled trials [14, 38, 40]. Bogani et al. [14] and Warlé et al. [40] did not observe a significant difference in visualization or progression, while Vijayaraghavan et al. [38] observed a significant decreased in visibility, visibility at suction, and space for dissection in the low-pressure PNP group when compared to standard pressure. Recent evidence indicates that the use of deep neuromuscular blockade may improve the incidence of optimal surgical space condition in laparoscopic cholecystectomy [72].

### Safety

With regard to serious adverse events and conversion to open procedure, no significant differences could be demonstrated for laparoscopic cholecystectomy [53, 73]. Recent RCTs comparing other laparoscopic procedures, e.g., laparoscopic hysterectomy, laparoscopic donor nephrectomy, and laparoscopic appendectomy, also indicate that low pressure has a comparable incidence of serious adverse events and conversions to open procedures when compared to standard pressure [14, 40, 74]. In all studies mortality was zero; however, it was only scarcely explicitly reported [16–19, 21, 27, 30, 40, 75].

## Discussion

Pain after laparoscopic procedures can be divided into three components: referred shoulder pain, superficial or incisional wound pain, and deep intra-abdominal pain [76]. Referred pain is most often attributed to CO_2_-induced diaphragm and/or phrenic nerve irritation causing referred pain to the C4 dermatoma, stretching of the diaphragm, and/or residual pockets of gas in the abdominal cavity [58, 77]. Deep intra-abdominal pain is mainly caused by bowel traction, stretch of the abdominal wall, and compression of intra-abdominal organs.

Although Gurusamy et al. [53] state that pain reduction does not always have clinical implications, there are several studies stating the importance of a clinically significant reduction in postoperative pain [54, 78]. Relative few number of blinded studies addressed postoperative pain after low-pressure PNP [22, 31, 38, 40]. However, in two of three blinded studies, a clinically relevant difference was found after 1 day. Only one blinded study assessed pain scores beyond 24 h and did not find a clinically relevant difference [40].

Overall inconsistency was minimal since in 15 [2, 9, 14, 16, 17, 22, 23, 31, 33, 38–41, 79, 80] of the 19 [2, 8, 9, 14, 16–18, 23, 24, 26, 28, 30, 33, 39, 41, 54, 79, 80] RCT’s a reduction in pain for low-pressure PNP was found. Reduction in pain scores ranged from 0.2 to 3.0 points on day 1. Except for 1 study [16], there were no studies reporting higher pain scores in patients who underwent low-pressure laparoscopy. Meta-analysis of pain scores showed significant less pain for low-pressure PNP, this difference was clinically relevant after 2 and 3 days.

The establishment of CO_2_ PNP increases intra-abdominal volume, thereby causing the diaphragm to move cranial. In combination with the fact that muscle relaxation during surgery impairs the excursion of the diaphragm, this can lead to compression of the lower lung lobes, resulting in increased dead space, ventilation perfusion mismatch, and decreased tidal volume [5, 7, 22, 25, 32, 51, 81]. Furthermore, CO_2_ is a highly soluble gas and is rapidly absorbed from the peritoneal cavity into the circulation. The resulting hypercapnia can only be avoided by compensatory hyperventilation. While low-pressure PNP was beneficial for the compliance of the lungs when compared to standard-pressure PNP, perioperative pulmonary parameters and postoperative pulmonary function tests are comparable, indicating that healthy individuals, with the aid of artificial ventilator adjustments, are able to compensate for pulmonary function reduction.

CO_2_ PNP can also have an impact on the cardiovascular system. Without preoperative volume loading, mechanical impairment of venous return as a result of inferior caval vein compression can result in reduced preload [37, 82]. Reduced preload can lead to decreased stroke volume and subsequent reduced cardiac output [83]. In addition, CO_2_ is absorbed in the systemic circulation, which can lead to hypercapnia and therefore stimulates the release of vasopressine and catecholamines and activates the renine–angiotensin–aldosteron system [84–86]. Vasopressine and catecholamines increase the systemic vascular resistance and therefore afterload [87, 88]. Furthermore, hypercapnia-induced acidosis can cause decreased cardiac contractility, sensibilization of myocardium to the arrhythmogenic effects of catecholamines, and systemic vasodilatation [89]. Due to these hemodynamic changes, invasive monitoring is necessary in ASA III and IV patients. These patients should also receive preoperative volume loading. In animal studies, low-pressure PNP is associated with improved cardiac function as compared to standard pressure, reflected by higher mean arterial pressure, cardiac output, and stroke volume [90–94]. However, in a human trial investigating ASA I and II patients, low-pressure PNP does not seem to have significant advantages when compared to standard-pressure PNP for cardiac function. However, no evidence exists regarding the beneficial effects of low pressure on cardiac function in ASA III and IV patients.

Transient elevation of liver enzymes such as AST and ALT after non-complicated cholecystectomy is a well-known finding [95]. This can be caused by cranial retraction of the gallbladder, cauterization of the liver bed, and manipulation of external bile ducts or effects of general anesthesia. However, elevated intra-abdominal pressure itself probably plays a significant part in elevation of liver enzymes. Since normal portal venous pressure is between 7 and 10 mmHg, increase in intra-abdominal pressure above this level reduces portal blood flow and may therefore cause a certain degree of hepatic ischemia [96–98]. Animal studies have shown a pressure-dependent decrease n hepatic blood flow, although this difference was not significant in all studies [93, 99, 100]. Likewise, postoperative AST and ALT were significantly increased when comparing low- versus standard-pressure PNP [101, 102]. For humans, the rise of AST and ALT seems to be related to intra-abdominal pressure, and this does not seem to apply for bilirubin, γ-GT, or ALP. For healthy patients, this is unlikely to have clinical consequences.

PNP is known to induce important changes in the kidneys. Increased intra-abdominal pressure can cause compression of the renal vessels and parenchyma. Reduced renal perfusion causes activation of the renin–angiotensin–aldosterone system, thereby further decreasing the renal blood flow. Also, several animal studies have reported elevated levels of antidiuretic hormone production (ADH) during increased intra-abdominal pressure, although the mechanism is poorly understood [85, 103]. Despite the fact that the studies were performed with a variety of animals and outcome measures, the results are uniform: Standard-pressure PNP is associated with decreased renal perfusion, urine output, postoperative creatinine, and creatinine clearance [6, 22, 83, 90, 104–109] when compared to low-pressure PNP. For humans, urine output was decreased in the standard-pressure group, but no changes in postoperative creatinine were observed.

No studies have been performed comparing the incidence of deep venous thrombosis in low- versus standard-pressure PNP. Observational studies in patients undergoing laparoscopic cholecystectomy with standard pressure have demonstrated a decrease in APTT and antithrombin III, suggesting activation of coagulation, and decrease in D-dimer, suggesting activation of fibrinolysis [8, 110–115]. Moreover, others have demonstrated an increase in peripheral vascular resistance and a decrease in flow rate in the leg during the PNP phase when standard-pressure PNP is used [116, 117]. Low-pressure PNP did not significantly alter thromboelastographic profile when compared to standard-pressure PNP [35].

The formation of postoperative peritoneal adhesions is an important complication following gynecological and abdominal surgery, having significant clinical and economic consequences. Surgery causes mesothelial defects, which produces an inflammatory exudate, resulting in the presence of a fibrin mass in the peritoneal cavity [118, 119]. When peritoneal fibrinolytic activity is normal, complete mesothelial regeneration occurs within 8 days. However, due to ischemia or inflammation-induced over-expression of plasminogen activator inhibitors 1 and 2, the peritoneal fibrinolytic activity can be suppressed, leading to incomplete removal of the fibrin mass from the abdominal cavity [120]. When fibrin persists, fibroblast migrates and organizes in adhesions [121].

The mechanism of adhesion formation as a consequence of increased abdominal pressure is unclear, but the most plausible explanation is hypoxemia caused by mechanical compression of the capillary bed. Possible effects of anoxaemia in the mesothelium include the induction of angiogenic factors, e.g., vascular endothelial growth factor [122] or attraction of monocytes from the circulation [123].

CO_2_ itself also seems to be an important factor in adhesion formation: adhesion formation decreased with the addition of 2–4 % oxygen [124, 125]. This can be explained by the fact that local hypercapnia induces acidosis and an impaired microcirculation [126, 127]. Two animal studies have been performed comparing adhesion formation in low- versus standard-pressure PNP. Rosch et al. [128] did not observe a difference in adhesion formation when comparing low- versus standard-pressure PNP after mesh implantation in chinchilla rabbits. On the contrary, Yesildaglar et al. [129] compared the adhesion scores in New Zealand rabbits following laser and bipolar lesions during endoscopic surgery and observed significant higher adhesion scores in the high intra-abdominal pressure group. Since Rosch et al. compared 3 versus 6 mmHg and Yesildaglar et al. compared 5 versus 20 mmHg, this might suggest that the significant difference observed by Yesildaglar et al. was caused by a greater pressure difference.

One human study suggests that low-pressure PNP minimizes the adverse effects on surgical peritoneal environment as measured by connective tissue growth factors, inflammatory cytokines, and cytotoxicity [49].

No human studies have been performed regarding the effects of low-pressure PNP on adhesion formation.

Anastomotic leakage continues to be a catastrophic complication of gastrointestinal surgery. Increased in-abdominal pressure diminishes intra-abdominal blood flow and could thereby impair the healing of anastomosis [130–132]. Animal studies have shown that anastomosis bursting pressure has an inverse correlation with intra-abdominal pressure [29, 133, 134]. However, it must be emphasized that some of the applied pressures are substantially higher than pressures that are normally used for laparoscopy. Moreover, in most studies the animals underwent open surgery via laparotomy after a period of abdominal insufflation, so the actual surgery on the intestines was performed after the PNP.

Intracranial pressure can be increased by elevated intraabdominal pressure. Increased intraabdominal pressure displaces the diaphragm cranially, thereby increasing intrathoracic pressure. This in turn leads to a reduction in venous drainage of the central nervous system, which causes an increase in cerebrospinal fluid and subsequently intracranial pressure [135–138]. In addition, absorption of carbon dioxide during the PNP phase can lead to hypercapnia, which causes reflex vasodilatation in the central nervous system and can therefore increase intracranial pressure [135].

Studies performed in swine indicate that there is a significant and linear increase in intracranial pressure with intraabdominal pressure [139].

Increase in intraocular pressure during laparoscopy is probably related to an increase in central venous pressure, caused by increased intrathoracic pressure [140–142]. Persistently increased intraocular pressure can lower ocular perfusion pressure and thereby cause progressive ischemic damage to the optic nerve. An animal study comparing the effect of low pressure (defined as 10 mmHg) to standard pressure (20 mmHg) in rabbits with α-chymotrypsin-induced glaucoma observed no significant increase in intraocular pressure after the start of PNP. However, intraocular pressure significantly increased with PNP in the head-down position, although it remained within the diurnal range [143]. A subsequent study did not observe any differences in terms of retinal layer organization and the distribution of intracellular vimentin and actin [144].

There are indications from animal studies that CO_2_ PNP is associated with tumor growth and metastases [145–147]. For instance, local and systemic hypercapnia reduces the phagocytic activity of macrophages, thereby stimulating growth of tumor cells [94, 148, 149]. Others suggested that increased intraabdominal pressure is associated with increased expression of genes associated with peritoneal tumor dissemination [150]. Results of animal studies comparing the development of liver and peritoneal metastases in low- versus standard-pressure PNP are inconclusive [151–157]. This can be explained by the used variety of animals, definition of low and standard pressure, and type of animal model. In most animal models, a tumor cell spillage model is used, in which cells are introduced at the time of surgery; however, this model does not reflect the clinical situation in which surgery is being performed on preexisting tumors.

IL-6 is a pro-inflammatory cytokine secreted by T cells and macrophages during infection and after tissue damage; CRP is an acute-phase protein that increases after IL-6 section. Both markers are an indication for the degree of tissue damage. Schietroma et al. suggested that low-pressure PNP was associated with significantly lower postoperative IL-6 and CRP. However, this could not be confirmed in four other studies.

PNP during laparoscopy is used to create workspace between the abdominal wall and intraabdominal organs. The major determinant of the amount of pressure that is required for adequate surgical conditions is the compliance of the abdominal wall. For example, in obese patients higher pressures are required to obtain adequate workspace and exposure of the surgical field. The compliance of the abdominal wall can be increased significantly by the application of a deep neuromuscular block. Furthermore, the use of deep neuromuscular block might increase intraabdominal space [158]. A recently performed systematic review suggests that the possible negative effects of low-pressure PNP on perioperative conditions might be overcome by the use of deep neuromuscular block, defined as PTC ≥ 1 to TOF 0, compared to moderate neuromuscular block [159].

All human studies included in this review switched directly from low to standard pressure in case of insufficient surgical conditions [9, 22, 24, 28, 30]. However, a stepwise increase in intraabdominal pressure guided by the quality of surgical field may be an ideal approach to identify the lowest possible pressure that is required to obtain adequate quality of the surgical conditions. Further research is required to investigate whether this approach leads to the use of lower intraabdominal pressures without compromising surgical conditions, and thus safety.

The design and implementation of the studies are the major limitations of this review. This was the main reason for downgrading the quality of evidence. Regarding cohort studies, all studies scored 4–7 points on the Newcastle–Ottawa scale. Also, it must be stated that the majority of the RCT’s was not registered in a trial registration.

## Conclusions and general recommendation (grade approach)

The first determinant of the strength of a recommendation is the balance between desirable and undesirable consequences of low-pressure PNP [160]. The use of low-pressure PNP decreases postoperative pain and analgesic consumption. With adequate pre- and perioperative measures, e.g., preoperative volume loading and artificial hyperventilation, the use of low- or standard-pressure PNP does not seem to have a major impact on cardiac or pulmonary functioning. Low-pressure PNP seems to improve peri- and postoperative dysfunction of liver and kidneys, although this is probably not clinically relevant for healthy patients. The effects of low-pressure PNP on thromboembolic complications, adhesions, tumor growth and metastases, intraocular, and intracranial need to be further specified. Until now, it is unclear whether low-pressure PNP procedures deteriorate surgical conditions; however, there does not seem to be an association with serious adverse events or conversion to open surgery. Regarding safety, Gurusamy et al. concluded that the safety of low pressure during laparoscopic cholecystectomy needs to be established [54]. Since the evidence for the use of low pressure during other laparoscopic procedures is limited, the general conclusion should be that safety of low pressure should be pursued in new clinical trials.

The second determinant is the quality of evidence, which is shown in Table 7. In general the quality of evidence was moderate to low.

**Table 7:** Summary of findings and quality of evidence regarding outcome measures that are potentially critical for decision making

Endpoints	Type of surgery (number of studies)	Outcomes	Quality of evidence
Pain	LC (15)	Less pain and lower overall analgesic consumption in the low-pressure group	B
	Other procedures (6)	Less pain in the low-pressure group	C
Pulmonary function	LC (4)	Although pulmonary compliance seems to be compromised in the standard-pressure group, this has little or no clinical consequences for ASA I and II patients.	B
	Other procedures(3)	One study describing decreased pulmonary compliance, no clinical consequences described in the other studies	B
Cardiac function	LC (4)	No differences between low and standard-pressure PNP for ASA I and II patients.	B
	LC (1)	No differences between low- and standard-pressure PNP for ASA III and IV patients.	B
	Other procedures (1)	No differences between low- and standard-pressure PNP.	C
Liver function	LC (6)	The rise of AST and ALT is related to intraabdominal pressure, although this is probably not clinically relevant for healthy individuals	B
	Other procedures (0)	No data	N/A
Kidney function	LC (0)	No data	N/A
	Other procedures(3)	Decreased urine output and clearance in the standard-pressure group, but no influence on postoperative creatinine after LDN	B
Thromboembolic complications	LC (3)	Inconclusive results	B
	Other procedures (1)	No significant difference in diameter of common iliac vein	B
Adhesions	Other procedures (0)	No data	N/A
Anastomosis healing	Colorectal procedures (1)	No data	N/A
Intracranial pressure	LC (0)	No data	N/A
	Other procedures (1)	PNP increases intracranial pressure in a pressure-dependent way	C
Intraocular pressure	LC	No data	N/A
	Other procedures	Pneumoperitoneum (standard pressure) increases intraocular pressure as compared to no pneumoperitoneum.	N/A
Tumor growth and metastases	LC	No data	N/A
	Other procedures	No data	N/A
Inflammation	LC	No significant difference in rise of pro-inflammatory cytokines (although not uniform results in all studies)	B
	Other procedures	Significant higher concentrations of IL-6, IL-1 and CRP in the standard pressure (1 study)	B
Visibility	LC (1)	Decreased visibility, decreased visibility at suction, decreased space for dissection	B
	Other (2)	No significant difference in difficulty or progression	B
Safety	LC (20)	No significant differences in incidence of serious adverse events or conversions to open surgery	B
	Other (3)	No significant differences in incidence of serious adverse events or conversions to open surgery	B

Quality of evidence and strength of recommendation were assessed according to the GRADE approach

A (high): Randomized trials; or double-upgraded observational studies

B (moderate): Downgraded randomized trials; or upgraded observational studies

C (low): Double-downgraded randomized trials; or observational studies

D (very low): Triple-downgraded randomized trials; or downgraded observational studies; or case series/case reports

Thirdly, values and preferences of physicians and patients regarding their attitude toward the use of low-pressure PNP and its potential beneficial effects have not been investigated.

The final determinant is costs. Decreasing intraabdominal pressure might prolong operation time and subsequently increase costs of the procedure. Indeed, in the Cochrane SRMA operation time was not significantly prolonged during laparoscopic cholecystectomy with low-pressure PNP (MD 1.51, 95 % CI 0.07–2.94, *I*^2^ = 0 %). In the same review, however, there was a tendency toward shorter hospital stay in the low-pressure group (MD −0.30, 95 % CI −0.63 to 0.02, *I*^2^ = 88 %) [53].

In summary, clinically the most important benefit of low-pressure PNP is lower postoperative pain scores. The cardiopulmonary consequences are comparable when for low- versus standard-pressure PNP in healthy patients; however, for ASA III and IV patients further studies are necessary. Moreover, safety of low-pressure PNP has to be established and the quality of evidence is moderate to low. Furthermore, no evidence exists on the value and preferences of physicians and patients regarding the potential benefits and risks of low-pressure PNP. Finally, there is no indication that the use of low-pressure PNP leads to increased healthcare costs. Altogether, we conclude that the recommendation to use low-pressure PNP is weak and that more studies are required to establish the safety of low-pressure PNP and to explore the values and preferences of physicians and patients.
